# Protective Effects of Meldonium in Experimental Models of Cardiovascular Complications with a Potential Application in COVID-19

**DOI:** 10.3390/ijms23010045

**Published:** 2021-12-21

**Authors:** Reinis Vilskersts, Dana Kigitovica, Stanislava Korzh, Melita Videja, Karlis Vilks, Helena Cirule, Andris Skride, Marina Makrecka-Kuka, Edgars Liepinsh, Maija Dambrova

**Affiliations:** 1 Laboratory of Pharmaceutical Pharmacology, Latvian Institute of Organic Synthesis, LV-1006 Riga, Latvia; stanislava.korzh@farm.osi.lv (S.K.); melita.videja@farm.osi.lv (M.V.); Karlis.Vilks@farm.osi.lv (K.V.); Helena.Cirule@farm.osi.lv (H.C.); makrecka@farm.osi.lv (M.M.-K.); ledgars@farm.osi.lv (E.L.); maija.dambrova@farm.osi.lv (M.D.); 2 Department of Pharmaceutical Chemistry, Faculty of Pharmacy, Rigas Stradins University, LV-1007 Riga, Latvia; 3 Department of Internal Diseases, Faculty of Medicine, Rigas Stradins University, LV-1007 Riga, Latvia; danakigitovica@gmail.com (D.K.); andris.skride@gmail.com (A.S.); 4 Department of Nephrology, Pauls Stradins Clinical University Hospital, LV-1012 Riga, Latvia; 5 Department of Molecular Biology, Faculty of Biology, University of Latvia, LV-1050 Riga, Latvia; 6 Department of Rare Diseases, Pauls Stradins Clinical University Hospital, LV-1012 Riga, Latvia

**Keywords:** mitochondria, COVID-19 cardiovascular complications, left ventricular dysfunction, right ventricular dysfunction, meldonium

## Abstract

Right ventricular (RV) and left ventricular (LV) dysfunction is common in a significant number of hospitalized coronavirus disease 2019 (COVID-19) patients. This study was conducted to assess whether the improved mitochondrial bioenergetics by cardiometabolic drug meldonium can attenuate the development of ventricular dysfunction in experimental RV and LV dysfunction models, which resemble ventricular dysfunction in COVID-19 patients. Effects of meldonium were assessed in rats with pulmonary hypertension-induced RV failure and in mice with inflammation-induced LV dysfunction. Rats with RV failure showed decreased RV fractional area change (RVFAC) and hypertrophy. Treatment with meldonium attenuated the development of RV hypertrophy and increased RVFAC by 50%. Mice with inflammation-induced LV dysfunction had decreased LV ejection fraction (LVEF) by 30%. Treatment with meldonium prevented the decrease in LVEF. A decrease in the mitochondrial fatty acid oxidation with a concomitant increase in pyruvate metabolism was noted in the cardiac fibers of the rats and mice with RV and LV failure, respectively. Meldonium treatment in both models restored mitochondrial bioenergetics. The results show that meldonium treatment prevents the development of RV and LV systolic dysfunction by enhancing mitochondrial function in experimental models of ventricular dysfunction that resembles cardiovascular complications in COVID-19 patients.

## 1. Introduction

Coronavirus disease 2019 (COVID-19) has become a global pandemic associated with significant mortality, mainly due to acute respiratory distress syndrome and fatal cardiovascular complications [1]. It has been shown that a substantial portion of hospitalized COVID-19 patients develop ventricular dysfunction [2]. The development of right ventricular (RV) and left ventricular (LV) dysfunction in COVID-19 patients has been associated with increased myocardial injury and mortality [3,4,5]. Usually, the development of RV [6] and LV [7,8] systolic dysfunction is linked to alterations in myocardial energy metabolism. In turn, the optimization of mitochondrial function in experimental models of RV [9,10] and LV [11] dysfunction improves ventricular function. COVID-19 patients present a significantly altered lipid metabolism, as evidenced by changed plasma and red blood cells levels of fatty acids (FAs) and their β-oxidation intermediates, acylcarnitines [12,13]. Moreover, COVID-19 patients with co-morbidities characterized by altered mitochondrial bioenergetics (e.g., diabetes and hypertension) have higher mortality [14,15]. Thus, the enhancement of mitochondrial functionality may be a promising strategy to improve the functioning of heart ventricles in COVID-19 patients and increase their survival options.

The etiological factors for the development of RV or LV dysfunction in COVID-19 patients still must be clarified; however, some recent studies have claimed that RV dysfunction develop due to severe lung damage, pulmonary hypertension and elevated levels of inflammatory cytokines [16]. In addition, it has been suggested that LV systolic impairment is driven by pronounced inflammatory burden and reduced myocardial supply [17,18]. Thus, agents that induce experimental RV or LV dysfunction through similar mechanisms can be used to study COVID-19-related ventricular dysfunction. Monocrotaline (MCT), a plant-derived alkaloid, induces RV failure by causing remodeling of pulmonary arteries and increasing pulmonary vascular resistance [19]. Moreover, administration of MCT induces lung inflammation [20] that is a pathogenic mechanism in COVID-19 patients. In addition, it has recently been shown that administration of lipopolysaccharide (LPS) causes an excessive immune response with subsequent deterioration of LV function [21]. Therefore, both agents induce ventricular dysfunction by mechanisms similar to those observed in viral infection and can be used to study COVID-19-related ventricular dysfunction.

Meldonium (3-(2,2,2-trimethylhydrazinium) propionate) is a clinically used cardioprotective drug that affects the bioavailability of L-carnitine long-chain acylcarnitines, regulates energy metabolism pathways and preserves mitochondrial function during myocardial ischemia-reperfusion [22,23,24]. In preclinical setups, meldonium decreases heart infarction size and attenuates the development of heart failure, arrhythmia, atherosclerosis, diabetes [22] and protects the liver against ischemia/reperfusion injury [25]. Meldonium has a good safety profile [26,27] and a long period of elimination in humans [28,29]. In clinical practice, meldonium is used in the complex therapy of chronic heart failure [30,31]. In addition, it has been shown that meldonium improves exercise tolerance in patients with stable angina [32]. Overall, these results suggest that treatment with meldonium can be beneficial for COVID-19-related cardiovascular complications.

This study was conducted to assess the effects of meldonium on experimentally induced RV and LV dysfunction, which resembles cardiovascular complications in COVID-19.

## 2. Results

### 2.1. Overall Animal Well-Being

The health of the animals was monitored every day, and none of the animals died during the four-week treatment. The weight gain of the animals that received MCT injection was slower than that of the control group animals (Figure S1); however, the weight gain in the MCT + meldonium group in the final week of the experiment was significantly faster than that in the MCT group. During the 28-day observation period, the weight gain in the animals in the control, MCT and MCT + meldonium groups was 86 ± 5, 26 ± 7 and 47 ± 4 g, respectively (Figure S1).

### 2.2. Effects of Meldonium on Ventricular Size and Function in the Right Ventricular Failure Model

Right ventricular systolic pressure was significantly increased in the MCT group compared to the control group, and treatment with meldonium did not attenuate the elevation in RV systolic pressure (Table 1). Administration of MCT induced the development of RV hypertrophy, which was evident by an elevated right ventricle-to-body mass index and Fulton index (right ventricle/(left ventricle+septum)). Treatment with meldonium attenuated the development of RV hypertrophy. As shown in Table 1, the administration of MCT did not increase the left ventricle-to-body mass index, and the size of the left ventricle was not influenced by treatment with meldonium.

**Figure 1:**
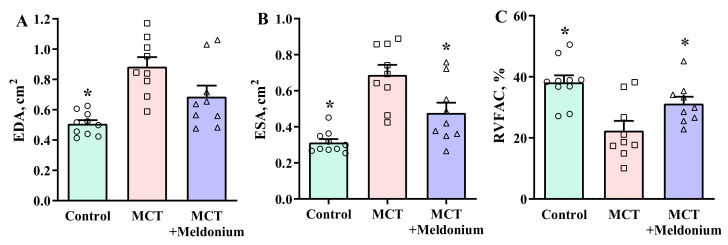
Effects of meldonium administration on the (**A**) end-diastolic area (EDA) of the right ventricle, (**B**) end-systolic area (ESA) of the right ventricle and (**C**) fractional area change of the right ventricle (RVFAC). Treatment with meldonium significantly decreased ESA and increased RVFAC compared with the monocrotaline (MCT) group. The data are shown as the mean ± SEM of 7 to 10 animals. * *p* < 0.05 vs. the MCT group, one-way ANOVA with Dunnett’s multiple comparison test.

Echocardiograms of the right ventricle from five animals in the MCT group and four animals in the MCT + meldonium group were excluded from the analysis due to poor echocardiogram quality, probably due to enlarged and fibrotic lungs that shadowed the heart during echocardiography. The analysis of echocardiographic parameters revealed that administration of MCT significantly increased the end-diastolic area (EDA) and end-systolic area (ESA) of the right ventricle (Figure 1A,B). Treatment with meldonium attenuated the development of dilatation of the right ventricle and significantly decreased ESA; moreover, the results showed a tendency of meldonium treatment to decrease the EDA compared with that of the MCT group. The EDAs in the control, MCT and MCT + meldonium groups were 0.5 ± 0.02, 0.9 ± 0.02 and 0.7 ± 0.07 cm^2^, respectively. The ESAs in the control, MCT and MCT + meldonium groups were 0.3 ± 0.02, 0.7 ± 0.06 and 0.5 ± 0.06 cm^2^, respectively. In addition, MCT administration significantly decreased the RV fractional area change (RVFAC) by 42%. Four weeks of treatment with meldonium significantly improved the functioning of the right ventricle and increased RVFAC by 40% compared with the MCT group (Figure 1C).

The analysis of the echocardiograms of the left ventricle demonstrated that 4 weeks after the administration of MCT, the rats had decreased end-diastolic volume, LV diameter at the end of diastole and cardiac output, although the ejection fraction and fractional shortening were unchanged (Table S1). Treatment with meldonium did not affect the dimensions or functioning of the left ventricle. In summary, these results indicate that treatment with meldonium attenuated the development of pulmonary hypertension-induced RV hypertrophy and failure.

### 2.3. Effects of Meldonium on Mitochondrial Function in the Right Ventricular Failure Model

**Figure 2:**
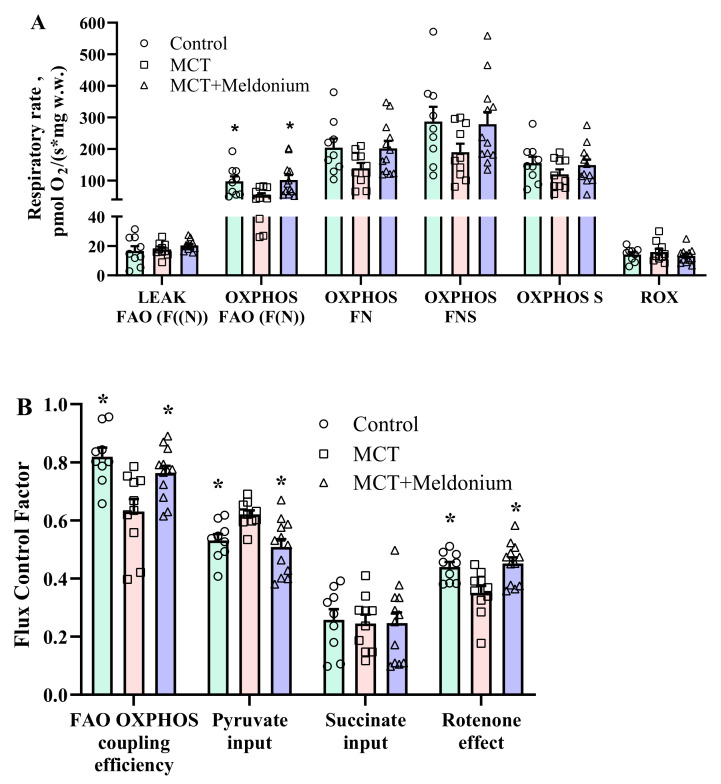
The effects of meldonium treatment (200 mg/kg for 4 weeks) on the mitochondrial respiration rate (**A**) and flux control factors (**B**) in right ventricular (RV) cardiac fibers 4 weeks after monocrotaline (MCT) injection. MCT administration induced inhibition of fatty acid (FA)-dependent oxidative phosphorylation, stimulation of pyruvate metabolism and partial complex I dysfunction. Treatment with meldonium restored mitochondrial functionality in the heart. The results are presented as the mean ± SEM of 9 to 12 animals. * *p* < 0.05 vs. the MCT group, one-way ANOVA with Dunnett’s multiple comparison test. Flux control factor, the contribution of each substrate/pathway to the respiration rate; S, succinate; F, FA oxidation-dependent pathway; N, NADH pathway; LEAK, substrate-dependent state; OXPHOS, oxidative phosphorylation-dependent state; ROX, residual oxygen consumption.

In the MCT group, FAO (F(N)) pathway-dependent respiration in the OXPHOS state was significantly decreased, by 46% (Figure 2A), which resulted in a 23% decrease in FAO-dependent OXPHOS coupling efficiency (Figure 2B) compared with that in the control group. Moreover, despite stimulation of pyruvate metabolism, as indicated by flux control factor analysis (Figure 2B), MCT administration decreased the FN and FNS pathway-linked respiration rates in the OXPHOS state (Figure 2A). In addition, in the MCT group, partial dysfunction of complex I was observed, as indicated by a decrease in flux control factor upon rotenone treatment (Figure 2B). Treatment with meldonium restored FAO-dependent OXPHOS coupling efficiency and subsequently restored pyruvate metabolism and prevented complex I dysfunction (Figure 2B). As a result, F(N), FN and FNS pathway-linked respiration in the OXPHOS state was improved in the MCT + meldonium group (Figure 2A). These results show that meldonium treatment normalizes mitochondrial function in the heart in conditions of pulmonary hypertension and RV failure.

### 2.4. Effects of Meldonium on Mitochondrial H_2_O_2_ Production after Anoxia-Reoxygenation in the Right Ventricular Failure Model

**Figure 3:**
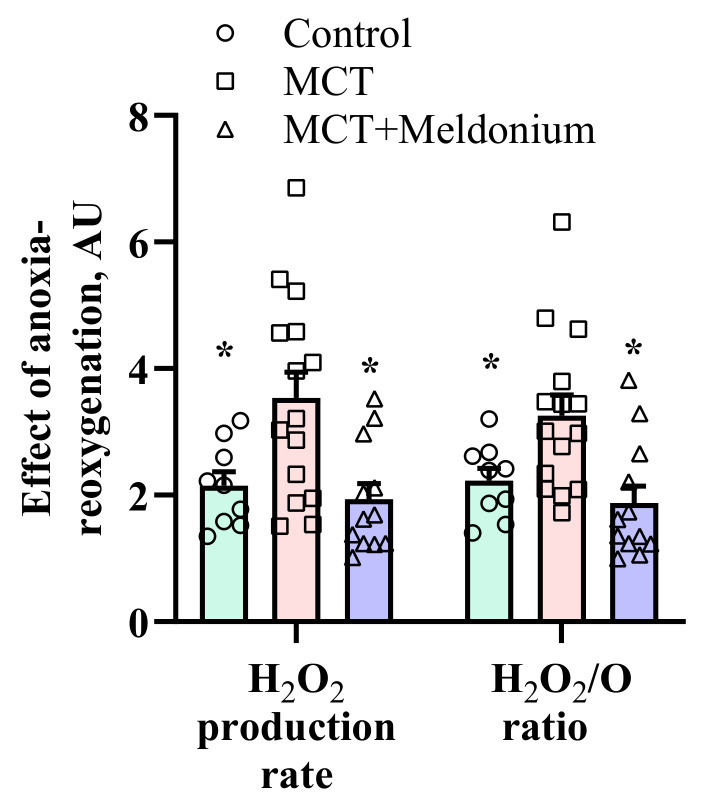
The effects of meldonium treatment (200 mg/kg for 4 weeks) on mitochondrial function/reactive oxygen species (ROS) production after in vitro anoxia-reoxygenation in left ventricular (LV) cardiac fibers in a model of pulmonary hypertension-induced right ventricle heart failure. Anoxia-reoxygenation induced a significantly higher H_2_O_2_ production rate and H_2_O_2_/O ratio in the monocrotaline (MCT) group than in the control group. Treatment with meldonium decreased anoxia-reoxygenation-induced H_2_O_2_ production to the level of the control group. The data are shown as the mean±SEM of 9 to12 experiments. * *p* < 0.05 vs. MCT group, one-way ANOVA with Dunnett’s multiple comparisons test.

The next step was to evaluate the effects of meldonium treatment on ischemia-reperfusion-related conditions. Mitochondrial functionality was evaluated after in vitro anoxia-reoxygenation in LV cardiac fibers. As shown in Figure 3, anoxia-reoxygenation induced an increase in the H_2_O_2_ production rate and H_2_O_2_/O flux ratio compared to normoxia in the control group. In the MCT group, the anoxia-reoxygenation-induced increases in the H_2_O_2_ production rate and H_2_O_2_/O ratio were 1.7- and 1.6-fold higher than those in the control group (Figure 3). Treatment with meldonium prevented the burst of H_2_O_2_ after anoxia-reoxygenation, as indicated by decreases in the H_2_O_2_ production rate and H_2_O_2_/O ratio (Figure 3), suggesting that meldonium protected cardiac mitochondrial function against ischemia-reperfusion-induced injury in conditions of pulmonary hypertension and RV failure.

### 2.5. The Effects of Meldonium on Lung Morphology, Endothelial Function and Blood Oxygen Saturation (SpO_2_) in a Right Ventricular Failure Model

The analysis of lung-to-body weight indexes revealed that MCT administration increased the lung weight of the animals two-fold (Figure S2). Treatment with meldonium for 4 weeks reduced the lung-to-body weight index by 16% (*p* < 0.05). The lung-to-body weight index in the control, MCT and MCT + meldonium groups was 3.0 ± 0.1, 6.4 ± 0.4 and 5.4 ± 0.3, respectively. Moreover, Masson’s trichrome-stained pulmonary slides revealed the development of massive fibrosis in the lungs of the animals in both the MCT and MCT + meldonium groups (Figure S3).

Two weeks after administration of MCT, dysfunction of the vascular endothelium and smooth muscle cells was observed in the rings of pulmonary arteries (Figure S4A,B). Treatment with meldonium did not improve MCT-altered vascular reactivity in pulmonary artery rings.

Before the administration of MCT, the average blood oxygen saturation was 96.3 ± 0.2% (Figure S5). A significant decrease in blood oxygen saturation was achieved only four weeks after the injection of MCT. Treatment with meldonium did not prevent a decrease in arterial blood oxygen saturation levels. The SpO_2_ level in the control, MCT and MCT + meldonium groups after 4 weeks of treatment was 95.6%, 94.2% and 94.1%, respectively. Administration of MCT or meldonium treatment did not affect the heart or respiratory rate.

### 2.6. Effects of Meldonium on Inflammation-induced Left Ventricular Dysfunction

**Figure 4:**
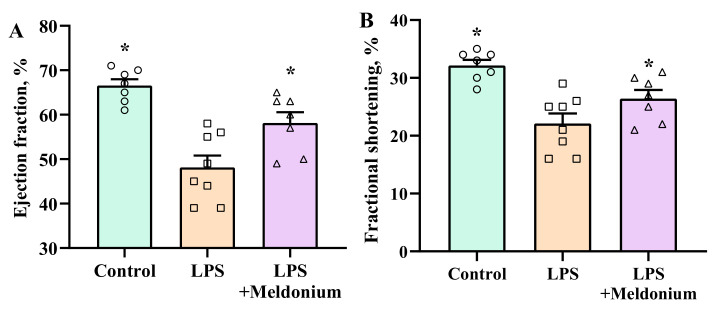
Effects of meldonium pretreatment on the lipopolysaccharide (LPS)-induced decrease in (**A**) ejection fraction and (**B**) fractional shortening. Pretreatment with meldonium significantly attenuated the development of the inflammation-induced decrease in the ejection fraction and fractional shortening of the left ventricle. The results are shown as the mean±SEM of 7 to 8 animals. * *p* < 0.05 vs. the LPS group, one-way ANOVA followed by Dunnett’s multiple comparison test.

Echocardiograms of two animals from the control and MCT + meldonium and one from MCT group were excluded from the analysis due to poor echocardiogram quality. Administration of LPS induced systolic dysfunction of the left ventricle, which was evident by decreased LV ejection fraction and fractional shortening (Figure 4). Pretreatment with meldonium significantly attenuated the inflammation-induced decrease in the ejection fraction and fractional shortening. Lipopolysaccharide-induced inflammation decreased the LV ejection fraction by 27%, but pretreatment with meldonium attenuated the decrease in the LV ejection fraction, and compared with the control group, it was reduced by 12%. In addition, fractional shortening in the LPS and LPS+Meldonium groups was decreased by 33% and 21%, respectively.

As shown in Table 2, administration of LPS significantly influenced the systolic dimensions of the heart, decreased intraventricular septal thickness at end-systole and LV posterior wall thickness at end-diastole and increased LV internal dimensions at end-systole and the end systolic volume. Pretreatment with meldonium attenuated the deterioration of the dimensions of the left ventricle. The aforementioned results indicate that treatment with meldonium prevents the deterioration of LV function that is caused by excessive inflammatory reaction.

### 2.7. Effects of Meldonium on Mitochondrial Function in Inflammation-Induced Left Ventricular Dysfunction

**Figure 5:**
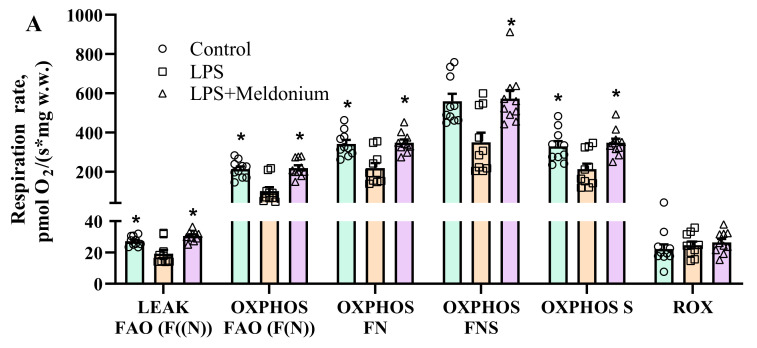

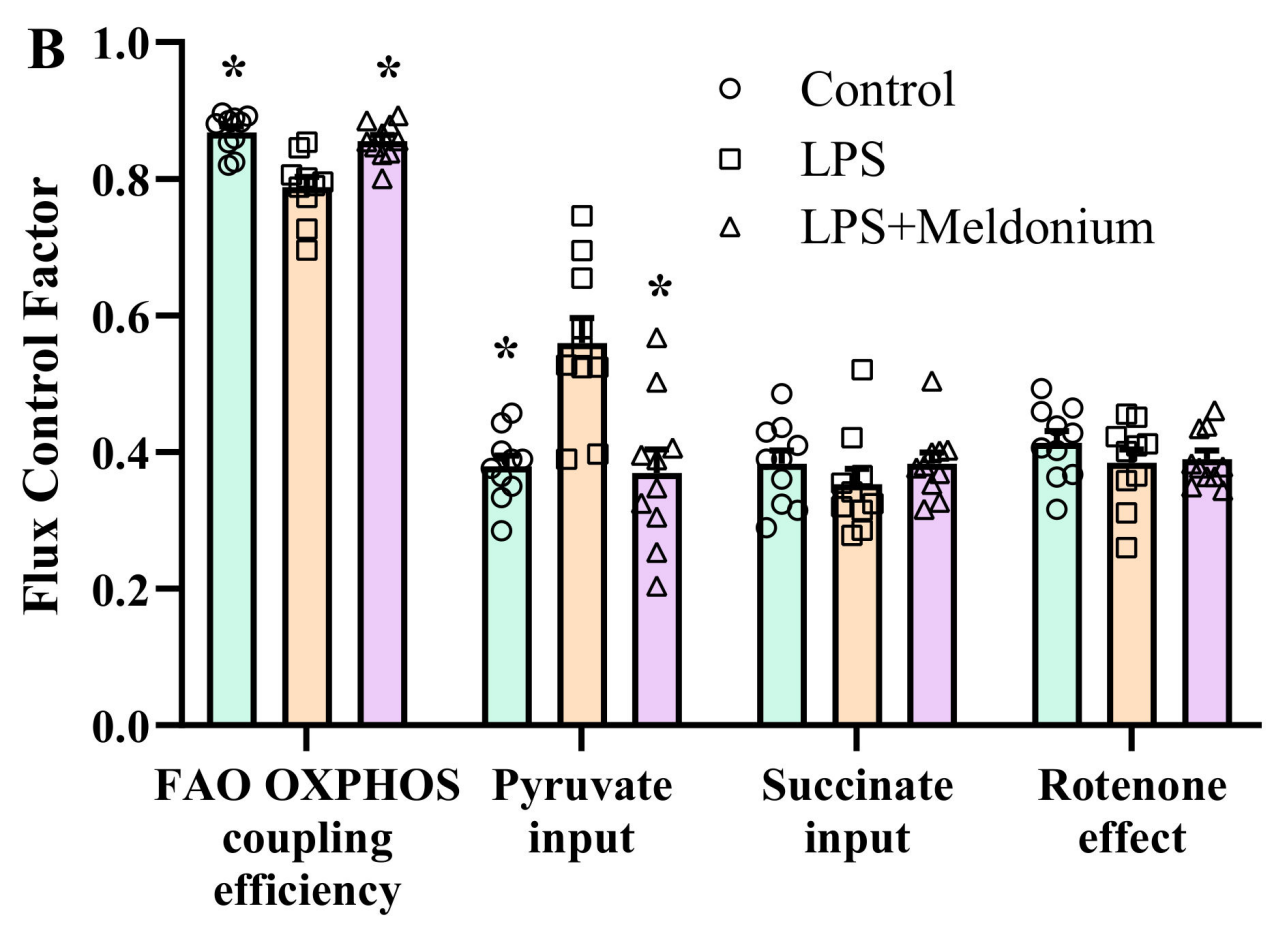
Mitochondrial respiration rate (**A**) and flux control factors (**B**) in cardiac fibers of the left ventricle after administration of meldonium at a dose of 100 mg/kg for 3 weeks in a model of lipopolysaccharide (LPS)-induced left ventricle dysfunction. LPS induced the inhibition of fatty acid (FA)-dependent oxidative phosphorylation (**A**,**B**) and subsequently increased pyruvate metabolism (**B**). Treatment with meldonium fully restored mitochondrial bioenergetics (**A**,**B**) to the level of the normal control group. The results are presented as the mean±SEM of 9 animals. * *p* < 0.05 vs. the LPS group, one-way ANOVA with Dunnett’s multiple comparison test. Flux control factor, the contribution of each substrate/pathway to the respiration rate; *p*, pyruvate; S, succinate; F, FA oxidation-dependent pathway; N, NADH pathway; LEAK, substrate-dependent state; OXPHOS, oxidative phosphorylation-dependent state; ROX, residual oxygen consumption.

Lipopolysaccharide administration inhibited F(N) pathway-linked respiration in the OXPHOS state by 53% (Figure 5A), which resulted in a 10% decrease in F(N) pathway-dependent OXPHOS coupling efficiency (Figure 5B) compared to the control group. Although LPS administration induced an increase in pyruvate metabolism without affecting other pathways (Figure 5B), LPS stimulation was not sufficient to restore the LPS-induced inhibition of respiration in the OXPHOS state (Figure 5A). Meldonium treatment prevented the LPS-induced decrease in FAO-dependent OXPHOS coupling efficiency and the subsequent upregulation of pyruvate metabolism (Figure 5B). Thus, in the LPS+ meldonium group, cardiac OXPHOS-dependent respiration was fully restored and comparable to that of the control group (Figure 5A). Overall, these results show that meldonium treatment preserves mitochondrial bioenergetics in the heart under conditions of acute inflammation.

Analysis of gene expression levels in mouse hearts revealed that administration of LPS markedly increased the mRNA levels of inflammatory factors *inducible nitric oxide synthase* (iNOS), *tumor necrosis factor alpha* (TNFα), *interleukin-1 beta* (Il-1β) and *interleukin-6* (Il-6). Treatment with meldonium did not reduce LPS-induced changes in inflammatory factor gene expression (Figure S6).

## 3. Discussion

In the present study, we demonstrate that treatment with meldonium attenuated the development of pulmonary hypertension-induced RV failure and inflammation-induced LV systolic dysfunction. The improvement of ventricular function in both models was attributed to improved mitochondrial bioenergetics. Taken together, these results suggest that treatment with meldonium attenuates the development of cardiovascular complications resembling those of COVID-19 by enhancing myocardial energy metabolism.

Ventricular dysfunction can be prevented by decreasing etiological factors or by directly stimulating ventricular contractility. It has been demonstrated that attenuation of RV remodeling and improvement of RV function can be achieved by reducing the increase in pulmonary vascular resistance [33] or by direct RV pharmacological stimulation by inotropic drugs [34]. Inflammation-induced LV dysfunction can be partly reversed by decreasing inflammation [35] or by using positive inotropic drugs [35]. Treatment with meldonium improved the functioning of pulmonary hypertension-induced RV failure and excessive inflammation-induced LV dysfunction; however, the enhanced contractility of the right ventricle was not related to endothelial function in pulmonary vessels, RV pressure or blood oxygen saturation. In addition, improvement of LV function was not related to a reduction in inflammation. This result indicates that meldonium is suitable for combination treatments with drugs that decrease pulmonary vascular resistance to reduce RV remodeling and improve RV function. Moreover, meldonium can be combined with anti-inflammatory drugs to improve heart function in cases of excessive inflammation and cytokine storm.

The development of RV failure is characterized by altered myocardial energy metabolism, such as downregulation of FA oxidation, altered oxidative metabolism and subsequent upregulation of glucose uptake and glycolysis [6]. In this study, we observed cardiac FA oxidation disturbances and complex I partial dysfunction after the development of RV failure. Fatty acid metabolism disturbances in the failing myocardium of the right ventricle have been attributed to the downregulation of peroxisome proliferator-activated receptor α/peroxisome proliferator-activated receptor-gamma coactivator 1alpha (PPARα/PGC1α) expression and decreased expression of several PGC1α target genes encoding key enzymes that regulate FA oxidation [36]. Previously, we showed that treatment with meldonium activated the PPARα/PGC1α pathway, increased the expression of genes involved in FA metabolism and stimulated mitochondrial β-oxidation [23]. In the present study, treatment with meldonium restored FA oxidation-dependent OXPHOS coupling efficiency in fibers obtained from the right ventricle of hearts with RV failure, which can be explained by activation of the PPARα/PGC1α pathway. In addition, treatment with meldonium decreased the accumulation of FA intermediates, thus facilitating the electron transfer system [37,38] and protecting mitochondrial function. It has been suggested that, at the mitochondrial level, loss of complex I assembly may be involved in the switching of energy metabolism to glycolysis [39]. Another study proposed that the alterations in mitochondrial function observed in RV failure can be mainly attributed to complex I dysfunction [40]. Treatment with meldonium reversed complex I dysfunction in the fibers of the myocardium of the right ventricle and thus restored the functionality of the electron transfer system. Overall, meldonium treatment maintained the function of the right ventricle due to the preservation of FA metabolism and complex I function.

The protective effects of metabolic modulators on altered energy metabolism and the function of the right ventricle have been studied previously [6]. Most of the previous studies have primarily focused on the activation of glucose metabolism by recoupling glycolysis with glucose oxidation due to inhibition of phosphorylation of the pyruvate dehydrogenase complex or indirectly by inhibition of FA metabolism [6]. A similar approach has also been used in cases of LV dysfunction and failure [41]. Meanwhile, there is experimental evidence indicating that the function of the failing left ventricle can be improved by stimulating myocardial FA metabolism [42]. Treatment with meldonium restored FA oxidation-dependent OXPHOS coupling efficiency to the level of healthy controls and improved the function of the right ventricle. Our results are the first to show that stimulation and restoration of decreased mitochondrial FA metabolism in the right ventricle is capable of improving the function of the ventricle. In contrast to stimulation of glucose oxidation, intensifying of FA oxidation would resemble more physiological energy metabolism in the right ventricle, as more than 70% of the ATP in the myocardium of the healthy right ventricle is produced by FA metabolism [43]. In the inflammation-induced LV dysfunction model, treatment with meldonium reversed FA oxidation and improved LV function but had no effect on inflammatory processes. Taken together, the results from both experimental models demonstrate that the restoration of mitochondrial bioenergetics is sufficient to improve ventricular function.

Mitochondria play critical role in cardiac ischemia-reperfusion injury because they are directly involved in ROS-producing pathophysiological mechanisms [44]. Overproduction of ROS can lead to cardiomyocyte damage and death [45]. Cardiac injury is common in hospitalized patients with COVID-19, and it has been revealed that in some patients, the damage is due to intracardiac thrombus and acute myocardial ischemia [46]. Our results show that treatment with meldonium attenuated ROS production in mitochondria after anoxia-reoxygenation, indicating preserved mitochondrial function that can result in decreased cardiac injury. Since it was previously shown that treatment with meldonium preserves mitochondrial functionality and decreases cardiac ischemia-reperfusion injury [47], it is suggested that meldonium treatment can also protect heart tissues of COVID-19 patients against ischemic damage.

RV failure predicts higher mortality not only in patients with COVID-19 [3,5] but also in patients with various chronic diseases characterized by elevated blood pressure in pulmonary arteries, valvular defects or injury of the myocardium of the right ventricle [48]. To date, few drugs have been available to directly stimulate the function/contractility of a failing right ventricle [48]. Meldonium has a good safety profile and long period of elimination in humans [28]. Some clinical studies have shown that treatment with meldonium enhances the function of the left ventricle in heart failure patients [30,31]. Our results suggest that administration of meldonium could improve RV function by modifying energy metabolism in the myocardium. Further research should be undertaken to test these preclinical findings in clinical trials.

In conclusion, our results show that meldonium treatment prevents the development of RV and LV systolic dysfunction and improves mitochondrial function in experimental models of cardiovascular diseases that resemble cardiovascular complications of COVID-19.

## 4. Materials and Methods

### 4.1. Materials

**Figure 6:**
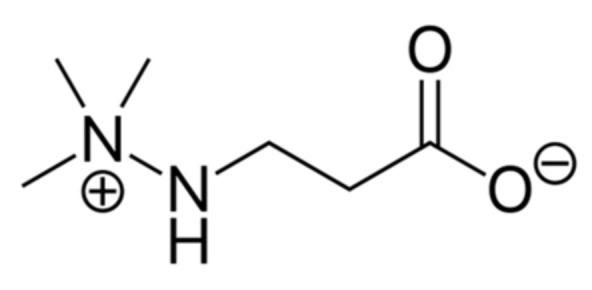
Chemical structure of meldonium.

Meldonium (Figure 6) was obtained from JSC Grindeks (Riga, Latvia). Isoflurane was obtained from Chemical Point Ltd. (Deisenhofen, Germany). All other reagents and chemicals were bought from the Sigma-Aldrich (St. Louis, MO, USA).

### 4.2. Animals

**Figure 7:**
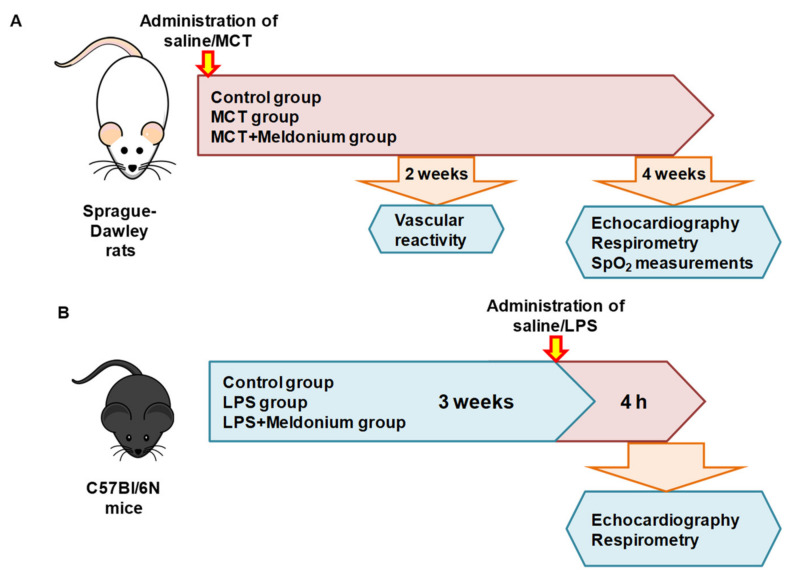
Schematic representation of the study design. MCT, monocrotaline; LPS, lipopolysaccharide.

Eight-week-old 100 male *Sprague–Dawley* rats were obtained from Charles River Laboratories (Sulzfeld, Germany), and thirty 8-week-old male *C57Bl6/N* mice were obtained from the Laboratory Animal Centre, University of Tartu (Tartu, Estonia). Animals were housed in individually ventilated cages (three rats per cage and five mice per cage) with unlimited access to food (R70 diet, Lantmännen Lantbruk, Sweden) and water. Standard housing conditions (temperature of 21–23 °C, 12-h light/dark cycle and relative humidity of 50 ± 5%). Rats and mice were adapted to these housing conditions for at least one week before the beginning of the experiments. The experimental procedures were performed in accordance with the guidelines of the European Community as well as local laws and policies, and the procedures were approved by the Latvian Animal Protection Ethical Committee of the Food and Veterinary Service, Riga, Latvia. All studies involving animals were reported in accordance with the ARRIVE guidelines [49,50]. To study the effects of meldonium on RV failure development, 34 rats were used. To study the effects of meldonium pretreatment on inflammation-induced LV dysfunction, 27 mice were used. To study the effects of meldonium on the development of endothelial dysfunction in pulmonary arteries, 36 rats were used and to study the effects of meldonium on blood oxygen saturation with experimental pulmonary hypertension models, 30 rats were used. The illustrative scheme of the experiments is displayed in Figure 7.

All experimental procedures and analyses were performed by a scientific staff blinded to the treatment groups and the experimental groups were uncovered only after summarizing results.

### 4.3. Pulmonary Hypertension and Right Ventricular Failure Model

Pulmonary hypertension and RV failure in rats were induced as described previously [51] with slight modifications. Pulmonary hypertension and RV failure were induced by a single subcutaneous injection of monocrotaline (MCT) at a dose of 60 mg/kg in 24 animals. Control group rats (*n* = 10) received an injection of an equal volume of saline. Rats that received MCT were randomly allocated to two equal groups (*n* = 12). The animals in the MCT group continued to receive purified water, while the rats in the MCT+ meldonium group started to receive meldonium at a dose of 200 mg/kg dissolved in purified water for four weeks. The weight of the animals was monitored twice per week after the administration of MCT or vehicle.

After 4 weeks of treatment, echocardiography and direct measurement of systolic RV pressure were performed. The rats were euthanized, and the pulmonary and cardiac tissues were harvested for histological analysis and assessment of mitochondrial functionality. The heart was cut out and divided into the right ventricle and the left ventricle with the septum. Both parts were weighted separately to calculate the Fulton index. Additional heart tissues from both ventricles were used to prepare permeabilized cardiac fibers to assess the functionality of mitochondria. The lungs were collected and weighed to calculate the lung-to-body weight index and prepared for histological analysis to assess the extent of fibrosis (see Supplementary Materials).

### 4.4. Echocardiographic Assessment of Cardiac Function in Rats

Echocardiography was performed using Philips iE33 ultrasonograph (Philips Healthcare, Andover, MA, USA) 28 days after the administration of MCT. The rats were anaesthetized with isoflurane (2%) dissolved in 100% oxygen. After the onset of anesthesia, the chest and upper part of the abdomen were shaved, and the animals were connected to the Philips ultrasound system to record the ECG from the second standard lead. Animals were placed on the left side, and an apical 4-chamber view was recorded to analyze the dimensions and functioning of the right ventricle using an S12-4 sector array transducer. Next, the animals were placed in the decubitus position. M-mode tracings of the left ventricle were recorded at the papillary muscle level with a linear L15-7io transducer.

### 4.5. Systolic Right Ventricular Pressure

After the echocardiographic parameters were recorded, the rats were intubated using a 16G intravenous catheter and artificially ventilated with a tidal volume of 1.5 mL/100 g animal [52] with 2% isoflurane dissolved in 100% oxygen. Median sternotomy was performed, and a 21G needle connected to a pressure transducer (ADInstruments) was inserted into the right ventricle and fixated while the RV systolic pressure reached a plateau.

### 4.6. Vascular Reactivity of Pulmonary Arteries

The reactivity of pulmonary arteries was assessed in isolated pulmonary arteries as described previously [53,54] with slight modifications. Twenty-four rats received subcutaneous injection of MCT at a dose of 60 mg/kg. Control group animals (*n* = 12) received an injection of an equal volume of saline. Rats that received MCT were randomly divided into two equal groups (*n* = 12). The animals from the first group (MCT group) continued to receive purified water, while the rats from the second group (MCT + meldonium) started to receive meldonium at a dose of 200 mg/kg dissolved in purified water for two weeks. Further animals were sacrificed and vascular reactivity assessed in pulmonary arteries. Experimental design more in details is described in Supplementary Materials.

### 4.7. Blood Oxygen Saturation (SpO_2_)

Thirty rats were divided into three equal-sized groups and were subjected to similar treatment as animals which developed pulmonary hypertension-induced RV failure. Before the administration of MCT and every week for next 4 weeks after administration of MCT, blood oxygen saturation was measured using pulse oximeter (MouseOx® Plus Pulse Oximeter for Rodents, Starr Life Sciences Corp., Oakmont, PA, USA). More detailed experimental design is described in Supplementary Materials.

### 4.8. Inflammation-Induced Left Ventricular Dysfunction Model

LV dysfunction in the mice was induced as described previously [14] with slight modifications. Before the experiment, the mice were randomly allocated to three groups (*n* = 9). Animals in the LPS + meldonium group started to receive meldonium at a dose of 100 mg/kg and purified drinking water. Animals from the two other groups (control and LPS groups) continued to receive purified drinking water. To induce experimental LV dysfunction after three weeks of treatment, the animals in the LPS and LPS + meldonium groups received intraperitoneal injection of LPS from Escherichia coli 0 55:B5 at a dose of 10 mg/kg. The control animals received an injection of saline. Four hours after LPS administration, the animals were subjected to echocardiography, and the mitochondrial function was measured in permeabilized cardiac fibers. The animals were sacrificed by decapitation, and heart tissue samples were collected. The heart samples were immediately used to assess mitochondrial function. In addition, part of the left ventricle was frozen in liquid nitrogen for PCR gene expression analysis (see Supplementary Materials).

LV dysfunction in the mice was induced as described previously [21] with slight modifications. Mouse model of inflammation-induced LV dysfunction instead of rat model was used as it is more appropriate to test scientific hypothesis in a proof-of-concept studies and it is used more frequently [55]. Before the experiment, the mice were randomly allocated to three groups (*n* = 9). Animals in the LPS + meldonium group started to receive meldonium at a dose of 100 mg/kg and purified water. Animals from the two other groups (control and LPS groups) continued to receive purified water. To induce experimental LV dysfunction after three weeks of treatment, the animals in the LPS and LPS + meldonium groups received intraperitoneal injection of LPS from Escherichia coli 0 55:B5 at a dose of 10 mg/kg. The control animals received an injection of saline. Four hours after LPS administration, the animals were subjected to echocardiography, and the mitochondrial function was measured in permeabilized cardiac fibers. The animals were sacrificed by decapitation, and heart tissue samples were collected. The heart samples were immediately used to assess mitochondrial function. In addition, part of the left ventricle was frozen in liquid nitrogen for PCR gene expression analysis (see Supplementary Materials).

### 4.9. Echocardiographic Assessment of Left Ventricle Functioning in Mice

The mice were anaesthetized using 5% isoflurane dissolved in pure oxygen. After the onset of anesthesia, the concentration of isoflurane was decreased to 2%, the experimental animals were placed in a decubitus position on a near-IR heating pad to maintain body temperature, and the chest was shaved. Commercially available depilation cream was used to remove remnants of fur. M-mode tracings of the left ventricle were recorded at the papillary muscle level using an iE33 ultrasound system (Philips Healthcare) equipped with a linear L15-7io transducer.

### 4.10. Mitochondrial Functionality Assessment

Mitochondrial function was assessed in permeabilized cardiac fibers that had been prepared as previously described [21,47]. Mitochondrial respiration was measured at 37 °C using an Oxygraph-2k respirometer (O2k; Oroboros Instruments, Innsbruck, Austria) in MiR05 medium (110 mM sucrose; 60 mM K-lactobionate; 0.5 mM EGTA; 3 mM MgCl_2_; 20 mM taurine; 10 mM KH_2_PO4; 20 mM HEPES, pH 7.1; and 0.1% BSA essentially free of FA).

The following protocol was used to evaluate mitochondrial functionality in both the MCT and LPS experiments. Palmitoylcarnitine and malate (10 µM and 0.5 mM, respectively) were added to measure fatty acid oxidation (FAO)-dependent mitochondrial respiration (F(N)-pathway) in the LEAK (L) substrate-dependent, state. Next, ADP was added at a concentration of 5 mM to initiate oxidative phosphorylation-dependent respiration (OXPHOS state). Then, pyruvate (5 mM, complex I substrate, N-pathway) was added to reconstitute FN pathway-linked respiration. Succinate (10 mM, complex II substrate, S-pathway) was added to reconstitute convergent FNS-linked respiration. Then, rotenone (0.5 µM, an inhibitor of complex I) and antimycin A (2.5 µM, an inhibitor of complex III) were added to determine the S-linked respiration and residual oxygen consumption (ROX), respectively.

To determine the contribution of each substrate to the respiration rate, the flux control factor was calculated as follows:

(1) $$1-\frac{\mathrm{Resp}.\mathrm{rate}\;\mathrm{before}\;\mathrm{the}\;\mathrm{addition}\;\mathrm{of}\;\mathrm{substrate}}{\mathrm{Resp}.\mathrm{rate}\;\mathrm{after}\;\mathrm{the}\;\mathrm{addition}\;\mathrm{of}\;\mathrm{substrate}}.$$

In addition, mitochondrial function during in vitro anoxia-reoxygenation was determined in permeabilized cardiac fibers prepared from the left ventricle of the MCT study animals as described previously [56,57]. Respiration measurements with simultaneous H_2_O_2_ flux detection were performed in MiR05 using an Oxygraph-2k respirometer. To induce anoxia, the maximal respiration rate of the sample was induced by the addition of substrates succinate (10 mM) with rotenone (0.5 µM) and ADP (5 mM), and the preparation was allowed to consume all the O_2_ in the respiratory chamber (within 10–20 min), thereby entering an anoxic state. After 30 min of anoxia, O_2_ was reintroduced to the chamber by opening the chamber. After 8 min of reoxygenation, the chamber was closed, and O_2_ flux was monitored for an additional 2 min. At the end of the experiment, antimycin A (2.5 µM) was added to determine the ROX level. H_2_O_2_ flux (ROS flux) was measured simultaneously by respirometry as described previously [58,59]. The effect of anoxia-reoxygenation-induced damage was calculated as the ratio from baseline values (i.e., from normoxia, the state before anoxia induction).

### 4.11. Data Analysis

All the data are expressed as the means ± SEM. Shapiro-Wilk normality test was used to assess the data distribution. One-way ANOVA with Dunnett’s multiple comparisons test was used for data that were distributed normally. Kruskal-Wallis with Dunn’s multiple comparison test was used for cases where the data were not distributed normally. Two-way repeated measures ANOVA with Tukey’s multiple comparison test was used to compare the differences in weight gain, vascular reactivity and blood oxygen saturation between the experimental groups. P values < 0.05 were considered to indicate statistical significance. The statistical calculations were performed using GraphPad Prism software.

## Figures and Tables

**Table 1:** Effects of meldonium administration on right ventricular (RV) systolic pressure, right ventricle-to-body, left ventricle-to-body mass indexes and Fulton index. Treatment with meldonium significantly attenuated the development of RV hypertrophy and had no effect on RV systolic pressure.

	Control	MCT	MCT + Meldonium
RV systolic pressure, mmHg	19 ± 1 *	52 ± 5	41 ± 4
Right ventricle-to-body mass index, mg/g	0.50 ± 0.01 *	1.13 ± 0.06	0.88 ± 0.08 *
Fulton index, g/g	0.27 ± 0.01 *	0.53 ± 0.03	0.43 ± 0.04 *
Left ventricle-to-body mass index, mg/g	1.8 ± 0.1	2.0 ± 0.1	2.0 ± 0.1

The results are shown as the mean±SEM of 10 to 12 animals. * *p* < 0.05 vs. the monocrotaline (MCT) group, one-way ANOVA with Dunnett’s multiple comparison test.

**Table 2:** Effects of meldonium administration on the dimensions of the left ventricle. Treatment with meldonium restored the systolic dimensions of the left ventricle after the injection of lipopolysaccharide (LPS).

	Control	LPS	LPS + Meldonium
IVSs, mm	1.1 ± 0.03 *	1.0 ± 0.03	1.1 ± 0.02 *
IVSd, mm	0.6 ± 0.02	0.5 ± 0.01	0.6 ± 0.02
LVPWs, mm	1.1 ± 0.04 *	0.8 ± 0.03	1.0 ± 0.04 #
LVPWd, mm	0.6 ± 0.04	0.5 ± 0.03	0.5 ± 0.02
LVIDs, mm	3.2 ± 0.1 *	3.6 ± 0.1	3.2 ± 0.1 *
LVIDd, mm	4.6 ± 0.1	4.5 ± 0.1	4.3 ± 0.1
ESV, mL	0.09 ± 0.01 *	0.11 ± 0.01	0.09 ± 0.01 *
EDV, mL	0.25 ± 0.02	0.23 ± 0.02	0.21 ± 0.01
HR, bpm	453 ± 27	501 ± 11	477 ± 10

Heart rate (HR), left ventricular posterior wall thickness at end-systole (LVPWs), left ventricular posterior wall thickness at end-diastole (LVPWd), interventricular septal thickness at end-systole (IVSs), interventricular septal thickness at end-diastole (IVSd), left ventricular internal dimension at end-systole (LVIDs), left ventricular internal dimension at end-diastole (LVIDd), end systolic volume (ESV) and end diastolic volume (EDV) of the animals from all three groups. The data are shown as the mean±SEM of 7 to 8 animals. # *p* < 0.07 vs. the LPS group, one-way ANOVA with Dunnett’s multiple comparison test, * *p* < 0.05 vs. the LPS group, one-way ANOVA with Dunnett’s multiple comparison test.

## Data Availability

The data underlying this article will be shared on reasonable request to the corresponding author.

## Supplementary Materials

The following are available online at https://www.mdpi.com/article/10.3390/ijms23010045/s1.

## Abbreviations

ACh	Acetylcholine
COVID-19	coronavirus disease 2019
EDA	end-diastolic area
EDV	end diastolic volume
ESA	end-systolic area
ESV	end systolic volume
FA	fatty acid
FAO	fatty acid oxidation
HR	heart rate
Il-1β	interleukin-1 beta
Il-6	interleukin-6
iNOS	inducible nitric oxide synthase
IVSd	interventricular septal thickness at end-diastole
IVSs	interventricular septal thickness at end-systole
LPS	lipopolysaccharide
LV	left ventricular
LVEF	left ventricular ejection fraction
LVIDd	left ventricular internal dimension at end-diastole
LVIDs	left ventricular internal dimension at end-systole
LVPWd	left ventricular posterior wall thickness at end-diastole
LVPWs	left ventricular posterior wall thickness at end-systole
MCT	monocrotaline
OXPHOS	oxidative phosphorylation-dependent respiration state
PGC1α	peroxisome proliferator-activated receptor-gamma coactivator 1α
PPARα	peroxisome proliferator-activated receptor α
ROS	reactive oxygen species
ROX	residual oxygen consumption
RV	right ventricular
RVFAC	right ventricular fractional area change
SNP	sodium nitroprusside
SpO_2_	oxygen saturation
TNFα	tumor necrosis factor alpha
