# Supplementary material for: Protective Effects of Meldonium in Experimental Models of Cardiovascular Complications with a Potential Application in COVID-19
Source: Int J Mol Sci. 2021 Dec 21;23(1):45. doi: 10.3390/ijms23010045 (PMC8744985; doi:10.3390/ijms23010045)
Supplement: Supplementary file 1 [file ijms-23-00045-s001.zip › ijms-1455679-supplementary.pdf]

Supplementary Materials

Expanded Materials & Methods

Histological preparations

Lungs from the rats were embedded in OCT freezing media, frozen and cut into serial 10-µm thick sections, and processed for staining. Masson's trichrome staining was performed to assess fibrosis in the lungs. The stained slides were analyzed under a Nikon Eclipse TE300 inverted microscope.

Vascular reactivity of pulmonary arteries

The reactivity of pulmonary arteries was assessed in isolated pulmonary arteries as described previously [53,54] with slight modifications. Twenty-four rats received subcutaneous injection of monocrotaline (MCT) at a dose of 60 mg/kg. Control group animals (n=12) received an injection of an equal volume of saline. Rats that received MCT were randomly divided into two equal groups (n=12). The animals from the first group (MCT group) continued to receive purified water, while the rats from the second group (MCT+Meldonium) started to receive meldonium at a dose of 200 mg/kg dissolved in purified water for two weeks. Rats were sacrificed by decapitation and exsanguination. The heart and lungs were removed en bloc, and the right and left extrapulmonary arteries were cleaned from surrounding tissues, dissected and cut into ~3 mm width rings. The obtained artery rings were mounted between two platinum hooks and incubated in organ baths in Krebs–Henseleit solution (composition (in mM): NaCl 118, $CaCl_2$ 2.5, $MgCl_2$ 1.64, $NaHCO_3$ 24.88, KH_2PO_4 1.18, glucose 10.0, and ethylenediaminetetraacetic acid 0.05), pH 7.4 at 37°C. The pulmonary artery rings were stretched to a resting tension of 0.5 g and equilibrated to the new conditions for 60 min. During the adaptation period, the incubation buffer solution was changed every 15 min. At the beginning of each experiment, the maximal contraction force of each ring was determined by adding 80 mM potassium chloride. Afterwards, the artery rings were washed until the resting tension was restored. After that, the pulmonary artery rings were precontracted with phenylephrine to 60%-80% of maximal contraction until a stable plateau contraction was reached. Endothelium-dependent relaxation was assessed by adding cumulative concentrations of acetylcholine (ACh) (10^{-9} to 10^{-5} mol/L). Furthermore, the pulmonary artery rings were washed until the resting tension was restored and once again precontracted with phenylephrine to 60%-80% of maximal contraction until a stable plateau contraction was reached. Endothelium-independent relaxation was assessed by adding cumulative concentrations of sodium nitroprusside (SNP) (10^{-10} to 10^{-5} mol/L).

Oxygen saturation in arterial blood

To study the effects of meldonium treatment on blood oxygen saturation in an experimental model of right ventricular (RV) failure, 30 male Sprague-Dawley rats were used. Twenty animals received subcutaneous injection of MCT at a dose of 60 mg/kg. Control group animals (n=10) received an injection of an equal volume of saline. Rats that received MCT were randomly divided into two equal groups (n=10). The animals from the first group (MCT group) continued to receive purified water, while the rats from the second group (MCT+Meldonium) started to receive meldonium at a dose of 200 mg/kg dissolved in purified water for two weeks. Blood oxygen saturation (SpO_2), respiratory rate and heart rate were measured before the administration of saline or MCT and once every week for 4 weeks using a pulse oximeter (MouseOx® Plus Pulse Oximeter for Rodents). Measurements were performed in awake and freely moving rats using a specialized collar (MouseOx - Collar clip Sensor), which was placed on the neck of the rat.

mRNA isolation and qPCR analysis

Total RNA from the mouse heart tissues was isolated using TRI reagent (Sigma-Aldrich), and first-strand cDNA synthesis was carried out using a High Capacity cDNA Reverse Transcription Kit (Applied Biosystems™, Foster City, CA) following the manufacturer's instructions. qPCR analysis of gene expression was performed by mixing SYBR® Green Master Mix (Applied Biosystems™) with synthesized cDNA and forward and reverse primers. Primers were designed using Primer-BLAST tool [59] and listed in Supplementary table 2 Reactions were run on a Bio-Molecular Systems MIC qPCR Cycler according to the manufacturer's protocol and using following conditions: 95°C for 10 mins, [95°C for 15 seconds, 60°C for 60 seconds] (60 cycles), 95°C for 60 seconds, followed by melt curve analysis 72-95°C, 0.3°C/sec. The relative expression levels for each gene were calculated with the ΔΔCt method and normalized to the expression of glucose-6-phosphate isomerase 1 (GPI1).

Supplementary results

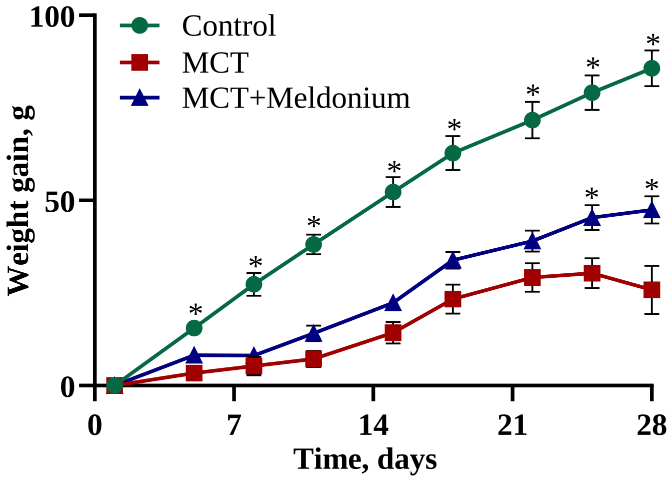

Figure S1. Effects of treatment with meldonium on the weight gain of the animals. Rats receiving meldonium gained weight significantly faster than animals from the monocrotaline (MCT) group. The results are shown as the mean ± SEM of 10-12 animals. * $p<0.05$ vs. MCT group, two-way repeated measures ANOVA with Tukey's multiple comparisons test.

Table S1. Effects of meldonium administration on the dimensions and functioning of the left ventricle. Treatment with meldonium had no effect on monocrotaline (MCT)-induced alterations in functioning of the left ventricle.

	**Control**	**MCT**	**MCT+Meldonium**
**IVSs, mm**	3.1±0.1	3.3±0.1	3.0±0.2
**IVSd, mm**	1.6±0.1	1.8±0.2	1.6±0.1
**LVPWs, mm**	3.2±0.1	2.9±0.1	3.0±0.1
**LVPWd, mm**	1.7±0.1	1.7±0.1	1.7±0.1
**LVIDs, mm**	4.9±0.1	3.9±0.3	4.3±0.5
**LVIDd, mm**	9.0±0.2*	7.2±0.5	7.7±0.5
**ESV, ml**	0.30±0.02	0.19±0.04	0.27±0.06
**EDV, ml**	1.55±0.09*	0.96±0.15	1.10±0.15
**EF, %**	81±3	82±4	80±10
**FS, %**	45±3	46±4	46±11
**HR, bpm**	310±8	298±8	305±6

Heart rate (HR), left ventricular ejection fraction (LVEF), fractional shortening (FS), left ventricular posterior wall thickness at end-systole (LVPWs), as well as stimulated left ventricular posterior wall thickness at end-diastole (LVPWd), interventricular septal thickness at end-systole (IVSs), interventricular septal thickness at end-diastole (IVSd), left ventricular internal dimension at end-systole (LVIDs), left ventricular internal dimension at end-diastole (LVIDd), end systolic volume (ESV) and end diastolic volume (EDV) of the animals from all three groups. The data are shown as the mean±SEM of 10 to 12 animals. * $p<0.05$ vs. the MCT group, one-way ANOVA with Dunnett's multiple comparison test.

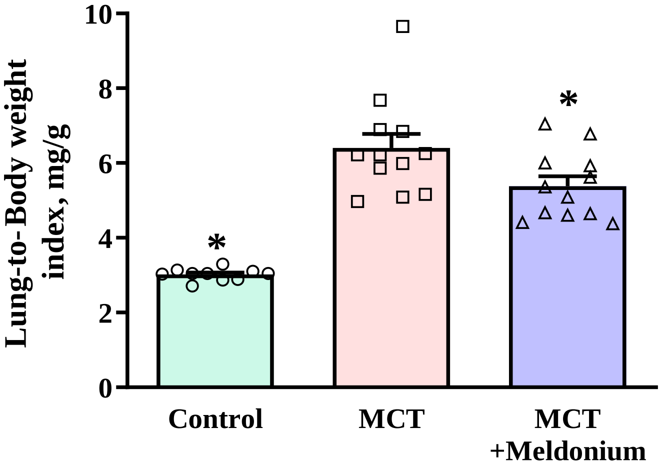

Figure S2. Effects of treatment with meldonium on the lung-to-body weight index. Four weeks after the administration of monocrotaline (MCT), animals from the MCT group had increased Lung-to-Body weight index. Treatment with meldonium decreased the elevated index. The data are shown as the mean ± SEM of 10 to 12 animals. * $p<0.05$ vs. the MCT group, one-way ANOVA with Dunnett's multiple comparison test.

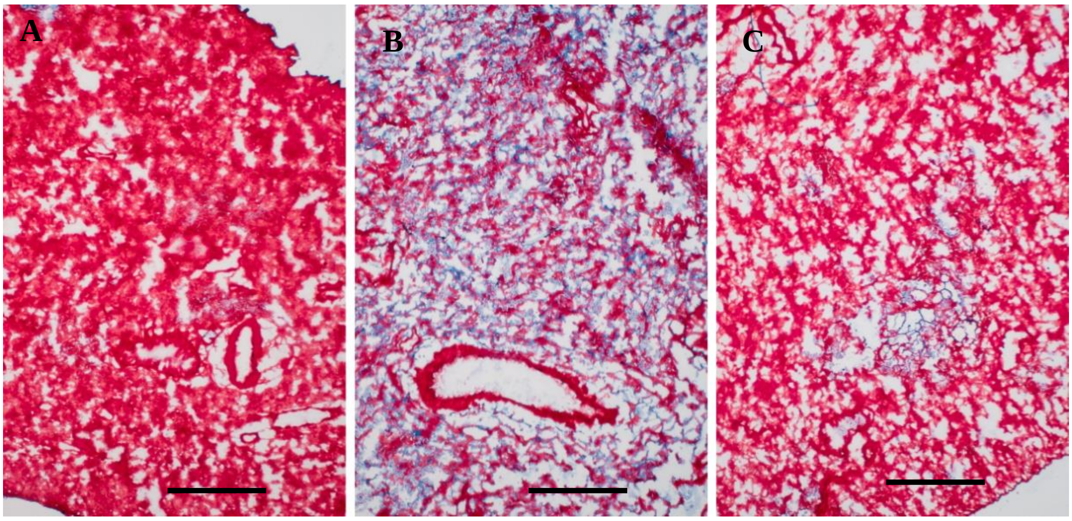

Figure S3. Representative images of Masson’s trichrome-stained sections of the lungs obtained from the (A) control, (B) monocrotaline (MCT) and (C) MCT+Meldonium groups. Tissues stained in red represent normal lung tissue, but the blue tissues are connective tissues that form lung fibrosis. Scale bar denotes 200 µm.

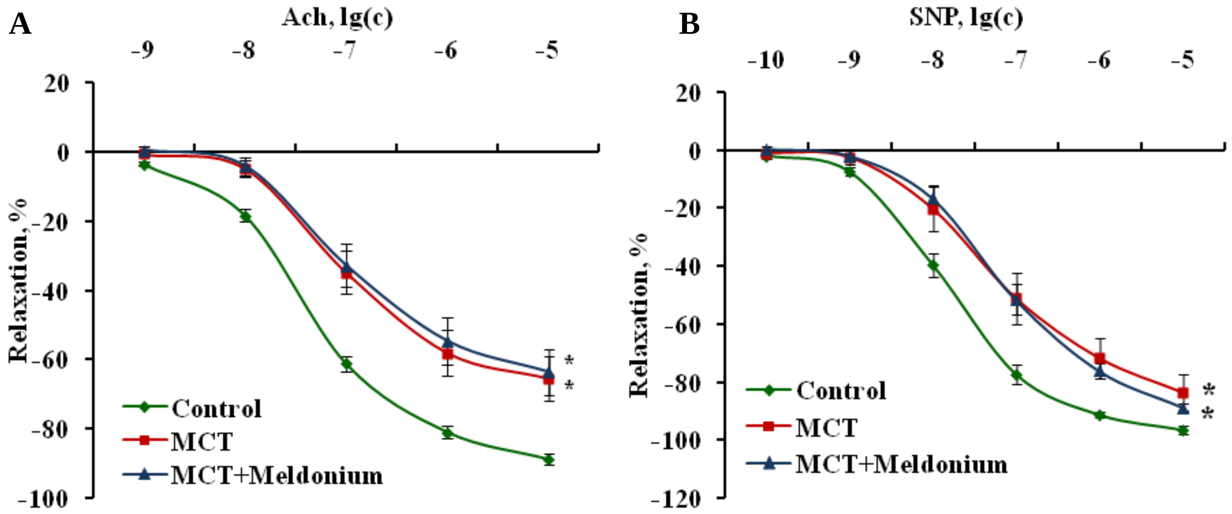

Figure S4. Effects of meldonium treatment on endothelium-dependent (A) and endothelium-independent (B) relaxation of pulmonary artery rings. Administration of monocrotaline (MCT) reduced endothelium-dependent and endothelium-independent relaxation of pulmonary artery rings. Treatment with meldonium had no effect on MCT-altered vascular reactivity. The results are shown as the mean±SEM of 12 rats. * p<0.05 vs. the MCT group, two-way repeated measures ANOVA with Tukey's multiple comparisons test. ACh, Acetylcholine, SNP; Sodium nitroprusside.

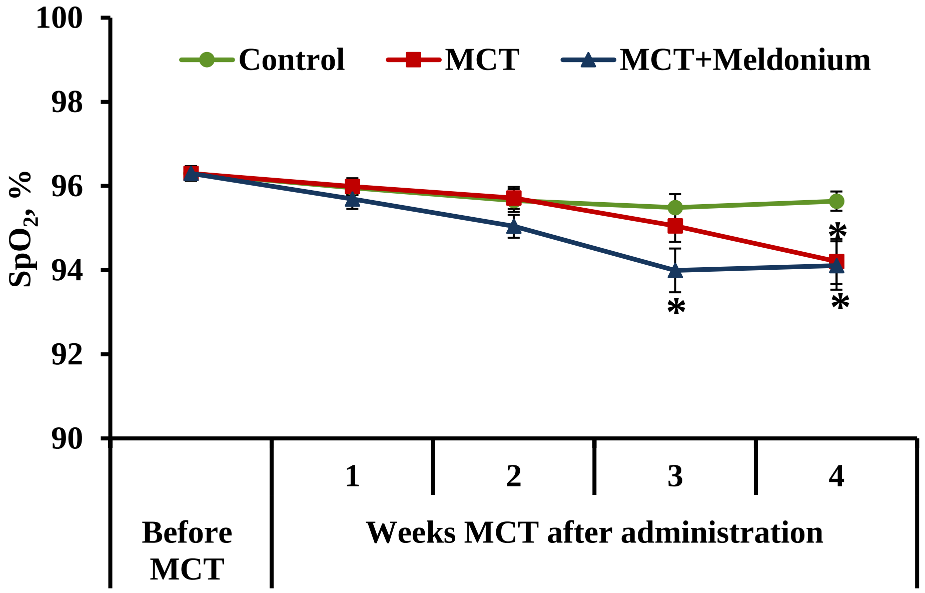

Figure S5. Effects of meldonium treatment on oxygen saturation (SpO_2) in the blood of awake and freely moving rats. Four weeks after administration of monocrotaline (MCT), a significant reduction in SpO_2 level was noted. Treatment with meldonium did not increase the SpO_2 level four weeks after administration of MCT. The results are shown as the mean±SEM of 10 rats. * p<0.05 vs. the control group, two-way repeated measures ANOVA with Tukey's multiple comparison test.

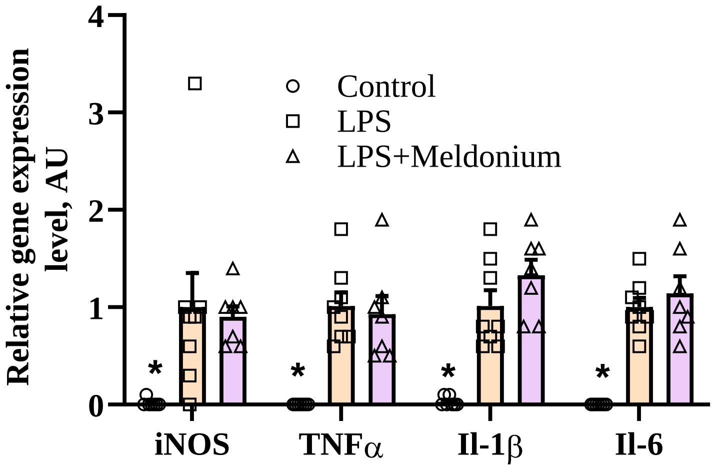

Figure S6. Effects of meldonium treatment on inducible nitric oxide synthase (iNOS), tumor necrosis factor alpha (TNFα), interleukin-1 beta (Il-1β) and interleukin-6 (Il-6) mRNA gene expression levels in heart tissues after administration of lipopolysaccharide (LPS) Administration of LPS significantly elevated the gene expression levels of the studied inflammatory genes. Treatment with meldonium had no effect on the expression of studied genes in the mouse myocardium after administration of LPS. The data are shown as the mean±SEM of 8 animals.
